# *Hedychiumviridibracteatum* X.Hu, a new species from Guangxi Autonomous Region, South China

**DOI:** 10.3897/phytokeys.110.28710

**Published:** 2018-11-02

**Authors:** Xiu Hu, Jia-qi Huang, Jia-chuan Tan, Yong-qingWu, Juan Chen

**Affiliations:** 1 Zhongkai University of Agriculture and Engineering, Zhongkai Road 501, Haizhu District, Guangzhou, Guangdong 510225, PR China Zhongkai University of Agriculture and Engineering Guangzhou China; 2 South China Botanical Garden, Chinese Academy of Sciences, Xingke Road, Tianhe District, Guangzhou, Guangdong 510650, PR China South China Botanical Garden, Chinese Academy of Sciences Guangzhou China

**Keywords:** *Hedychium*, Zingiberaceae, new species, Guangxi Autonomous Region

## Abstract

*Hedychiumviridibracteatum* X.Hu, a new species from Guangxi Zhuang Autonomous Region, South China, is described and illustrated. *Hedychiumviridibracteatum* X.Hu is included in the short-anther group of *Hedychium* and is most similar to HedychiumvillosumWall.var.tenuiflorum Voigt ex Baker, H.villosumWall.var.villosum Wall., and *H.chingmeianum* N. Odyuo & D. K. Roy.

## Introduction

*Hedychium* J. Koenig, 1783 (Zingiberaceae, Zingibereae) is a genus with about 50–80 species mainly distributed in tropical Asia (Jain and Prakash 1995, Wu and Larsen 2000). With pleasant fragrances and beautiful flower forms, *Hedychium* species are used as cut flowers or to decorate landscapes.

Twelve species of *Hedychium* have been reported in Guangxi Zhuang Autonomous Region, South China (Wu and Larsen 2000). According to Wu and Larsen (2000)*Hedychiumvillosum*, has two varieties, *H.villosum* Wall. with large flowers and H.villosumvar.tenuiflorum Wall. ex Baker with small flowers. Yu et al. (2010) proposed that the two varieties should be recognised as distinct species on the basis of their discernible morphological characteristics, different polyploid levels and flowering time, which contribute to complete reproductive isolation. Sanoj et al. (2013), having compared the original specimens and descriptions, concluded that the plant with small flowers was the true H.villosumWall.var.villosum Wall., and the one with large flowers was H.villosumWall.var.tenuiflorum Voigt ex Baker. Concurring with Yu et al. (2010), Gao et al. (2014) referred to H.villosumvar.tenuiflorum Wall. ex Baker (Wu and Larsen 2000) as an independent species, *H.tenuiflorum* (Baker) K. Schum. In this paper, the treatment of *H.villosum* Wall. and its related species follows Sanoj et al. (2013).

In the autumn of 2008 we received a flowering plant of *Hedychium* from a local person in Napo, Guangxi Autonomous Region, South China. The plant resembled H.villosumWall.var.tenuiflorum (Wall. ex Voigt) Wall. ex Baker (Sanoj et al. 2013) in having long filaments and sagittate anthers but could be distinguished from the above by its pure white flower, broad leaves, short ligule, short green bracts and bracteoles. After a further field investigation, morphological studies, flow cytometric analysis of nuclear DNA content, and comparison with published protologues and descriptions (Baker 1890–1892; Schumann 1904; Schilling 1982; Ridley 1898; Larsen 1965; Jain and Prakash 1995; Wu and Larsen 2000; Pornpimon 2008; Yu et al. 2010; Sanoj et al. 2013; Odyuo and Roy 2017), the plant in question was confirmed to be a new species of *Hedychium*.

## Materials and methods

### Morphological comparison and distribution mapping

In October 2011 we carried out a field survey and found the new plant in the wild in the vicinity of the Nongyi village, Napo County, Guangxi Zhuang Autonomous Region. The new plant grows on steep rocks under the forest canopy, at about 600 m a.s.l. Before that field trip, we had collected *Hedychiumvillosum* Wall. in the field from different part of China between 2006 and 2010. Based on those field surveys and collections, the affinities between the new species and its relatives, *H.villosum* Wall., were compared to living plants and specimens. Guided by the collection site of several suspected specimens, we made further field investigations after 2012 to gain a better understanding of the distribution range of the new plant. By combining the geographical distribution data collected from field investigations, specimens (GH, GXMI, HITBC, IBK, IBSC, KUN, PE) and references (Yu et al. 2010), we made a distribution map of the new plant and the related species using DIVA-GIS (version 7.5) (http://www.diva-gis.org/).

### Ploidy level analysis of the new plant and its related species

Flow cytometric analysis of nuclear DNA content of the new species and its relatives was performed according to Sakhanokho et al. (2009). Ploidy levels were determined for young leaf tissues. A piece of leaf tissue of about 1 cm^2^ was chopped with a sharp razor blade in a petri dish containing 0.4 ml nuclei extraction buffer. After a 3 min incubation with gentle agitation, the extract was poured through a 50 µm mesh sieve. DNA fluorochrome, a nucleus staining buffer, was added to the extract buffer in the ratio of 4:1 and the sample was analysed immediately for the DNA content of the nuclei. Buffers were supplied as part of the Cystain ultraviolet Precise T reagent kit (Partec GmbH, Münster, Germany). The fluorescence of the nuclei was measured using a Partec Cy Flow Space flow cytometer (Partec GmbH, Münster Germany). Sample measurements were replicated three times for each plant. The results were displayed as histograms showing the number of nuclei grouped in peaks of relative fluorescence intensity, which is proportional to the DNA content. To determine the standard peak of diploid cells (2C DNA), leaf tissues were collected from young leaves of diploid *Hedychiumcoronarium* plants (Hu et al. 2011; Ramachandran 1969). The instrument gain was adjusted so that the peak of nuclei isolated from diploid (*H.coronarium*) was set at channel 50 and this calibration was checked periodically to minimise variation resulting from runs. Therefore, peaks representing nuclei from samples with diploid and tetraploid levels were expected at channels 50 and 100, respectively.

## Results

### Hedychium viridibracteatum

Taxon classificationPlantaeZingiberalesZingiberaceae

X.Hu sp. nov.

urn:lsid:ipni.org:names:77191580-1

Figures 1 , 2 , 3

#### Diagnosis.

*Hedychiumviridibracteatum* X.Hu, sp. nov. is morphologically similar to H.villosumWall.var.tenuiflorum Voigt ex Baker by having sagittate anther, long filament, relatively thick and small leaves, more than two flower per-bract, but can be easily distinguished from it by its green (vs. brown) shorter bracts (1.3–1.5 × 0.4–0.5 cm vs. 2.7–2.9 × 1.1–1.2 cm) and bracteoles (1.0–1.1 × 0.3–0.4 cm vs. 2.1–2.2 × 0.7–0.75 cm), pure white flowers (vs. white with red stamen), dentate (vs. acute) tips to the lateral staminodes, and the apex of the labellum incised to the middle (vs. deeply divided).

#### Type.

China, Zhuang Autonomous Region, Napo county, Nongyi village, on rocks, 22°57.727'N, 106°00.265'E, alt. 696 m, 6 October 2011, *Xiu Hu* 267 (holotype: IBSC; isotype: MO).

#### Description.

Epilithic, evergreen perennial rhizomatous herbs. Rhizome purplish brown outside, sheathed, creamy white inside, 2–4 cm in diameter. Leaf stems slender, 60–100 × 0.5–0.7 cm, glabrous, 7–10 leaves. Ligule oblong-ovate, green or purple, glabrous, 1.8–2.3 cm × 1.2–1.5 cm, membranous. Leaf blades elliptic,15–25 × 5–8 cm, base attenuate into a short petiole, 0.5 cm long, apex caudate, glabrous on both sides and dark green above, purple beneath, especially the leaves in the lower part of the stem. Inflorescence a terminal spike, erect, 10–15 cm long, lax flowered; peduncle 1.5–2.0 cm long, green; rachis green, pubescent. Bracts oblong-ovate, apex acute, green, 1.3–1.5 × 0.4–0.5 cm, coriaceous, pubescent outside, glabrous inside, 2–4 flowered. Bracteoles tubular, apex acute, green, coriaceous1.0–1.1 × 0.3–0.4 cm, pubescent outside, glabrous inside. Flowers pure white, 10–11 cm long, lightly fragrant. Calyx tubular, green, 2.5–2.8 × 0.12–0.15 cm, apex obtusely 3-toothed, pubescent. Floral tube pure white, 4.0–5.0 cm long, slender; lobes glabrous, pure white, reflexed, linear, dorsal lobe 3.1–3.3 × 0.12 cm, lateral lobes 2.9–3.1 × 0.12 cm. Lateral staminodes linear-ridged, pure white, 3.0–3.3 cm long, dentate at apex. Labellum ovate, pure white, 2.8–3.0 × 1.2–1.4 cm, base attenuate into a claw, 0.8–1.0 × 0.2 cm, apex incised to the middle, white. Filament 6.0–6.8 cm long, white; anther dorsifixed, sagittate, 3.5–4 mm long, glabrous, light yellow. Ovary 0.4–0.5 × 0.2–0.4 cm, densely silky hairy, trilocular, placentation axile; epigynous glands 2, slender, ca. 0.2 cm long, yellow. Stigma green, ciliate. Tetraploid.

**Figure 1.**
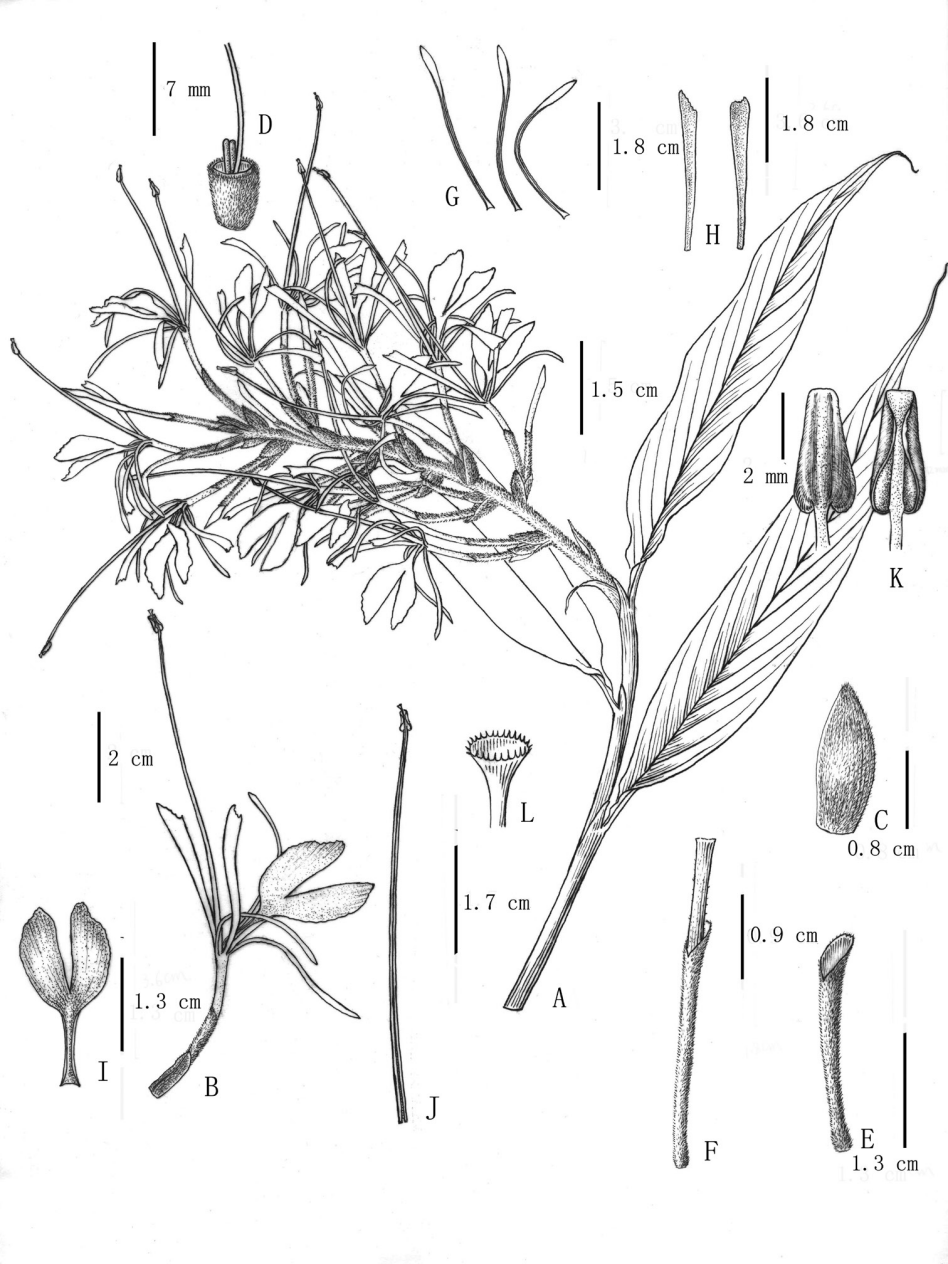
*Hedychiumviridibracteatum* X.Hu, sp. nov., holotype, **A** upper leaves and inflorescence **B** flower **C** bract **D** ovary and glands **E** calyx tube **F** floral tube with calyx tube wrapped outside **G** corolla lobe **H** lateral staminodes **I** labellum **J** stamen **K** anther **L** stigma. Drawings Y. X. Liu.

**Figure 2.**
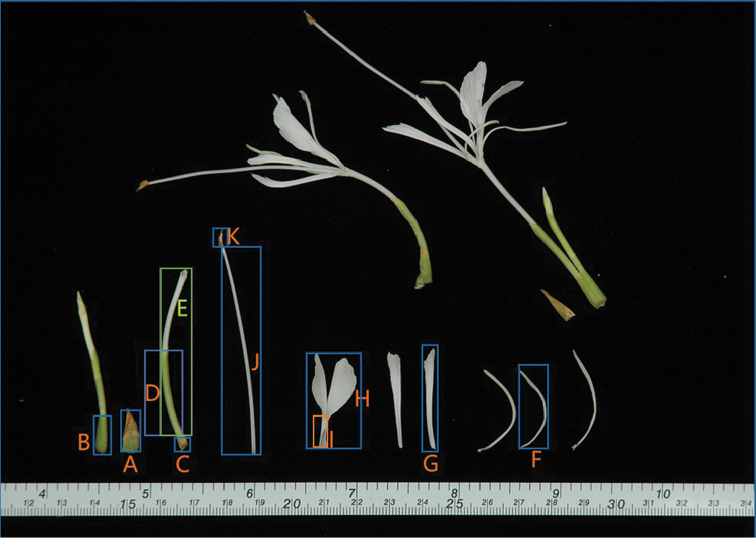
*Hedychiumviridibracteatum* X.Hu, sp. nov., flowers and their parts: **A** bract **B** bracteole **C** ovary **D** calyx tube **E** floral tube **F** corolla lobe **G** lateral staminodes **H** labellum **I** labellum claw **J** filament **K** anther.

**Figure 3.**
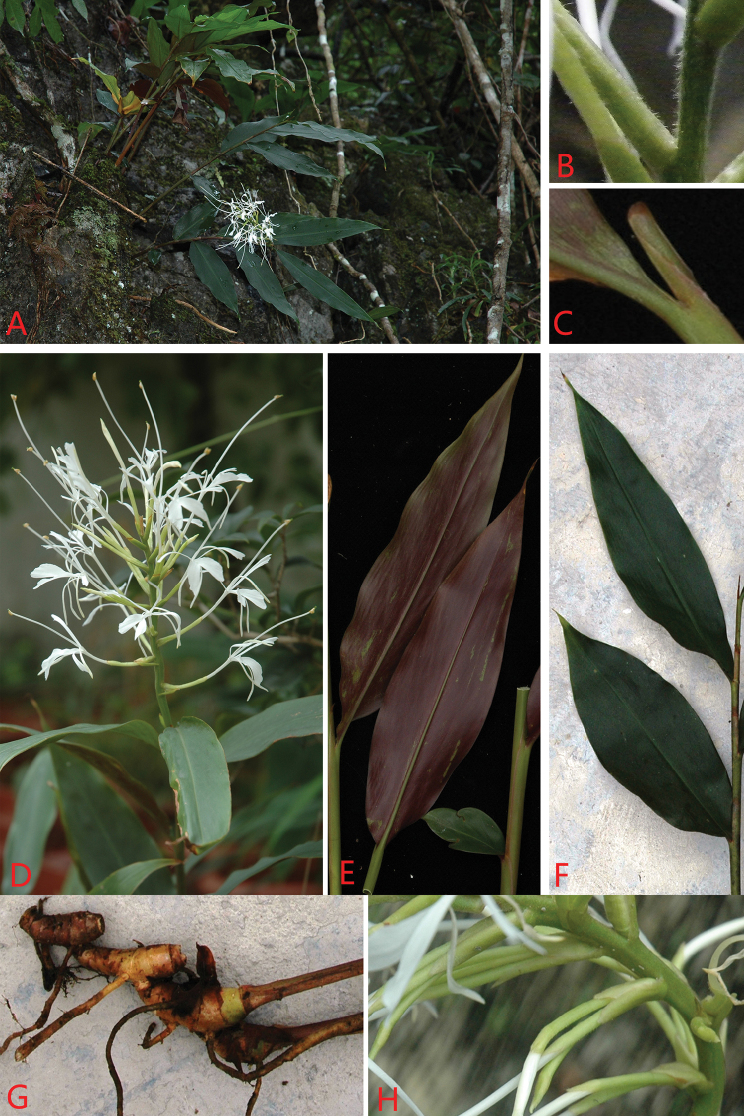
*Hedychiumviridibracteatum* X.Hu sp. nov. **A** habit (growing on rocks) **B** rachis and bracts (pubescent) **C** ligule (glabrous) **D** inflorescence at anthesis **E** leaf blade surface (adaxial view) **F** leaf blade surface (abaxial view) **G** rhizome **H** portions of inflorescence (2–4 flowers per bract).

#### Flowering.

September–October.

#### Fruiting.

Unknown.

#### Etymology.

The new species is named after its green bracts and pure white flowers which are highly diagnostic.

#### Habitat.

This species is currently found on limestone rocks in Guangxi Autonomous Region (Napo, Longzhou and Jingxi Counties) mainly growing under forest at altitudes of 600–800 m.

#### Ploidy level analysis.

**Figure 4.**
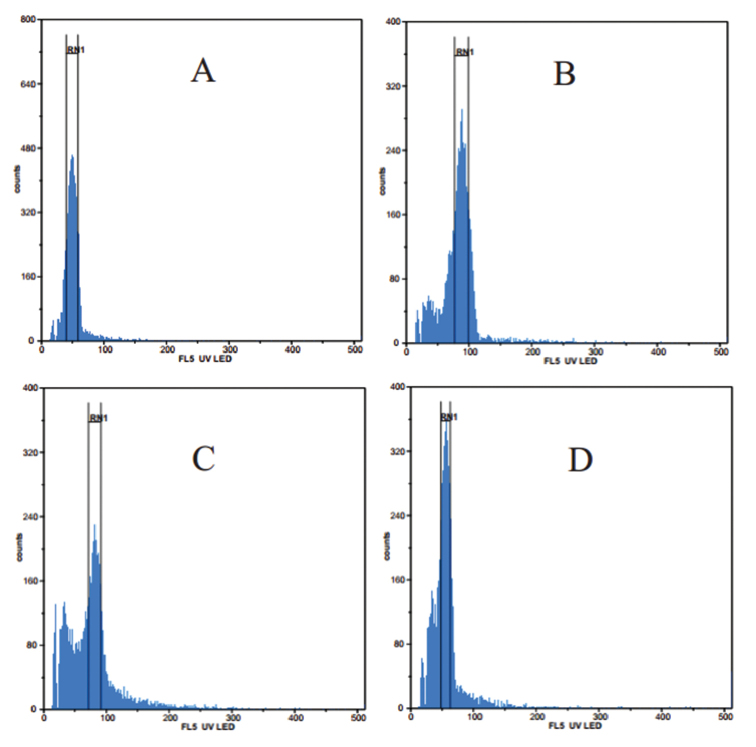
Histogram of relative DNA content of nuclei isolated from the leaves of the new plant and related *Hedychium* species. **A***H.coronarium* (control) **B***H.viridibracteatum***C**H.villosumvar.tenuiflorum**D**H.villosumvar.villosum.

The results (Fig. 4) show that, when the nuclei of the diploid control (Fig. 4A), *Hedychiumcoronarium* (Hu et al. 2011; Ramachandran 1969) were set to channel 50, the new plant, *H.viridibracteatum* X.Hu (Fig. 4B) resolved at channel 100. This means that the new plant is a tetraploid which shares the same level of ploidy with H.villosumWall.var.tenuiflorum Voigt ex Baker (Fig. 4C) but differs from H.villosumvar.villosum Wall. (Fig. 4D).

#### Distribution.

**Figure 5.**
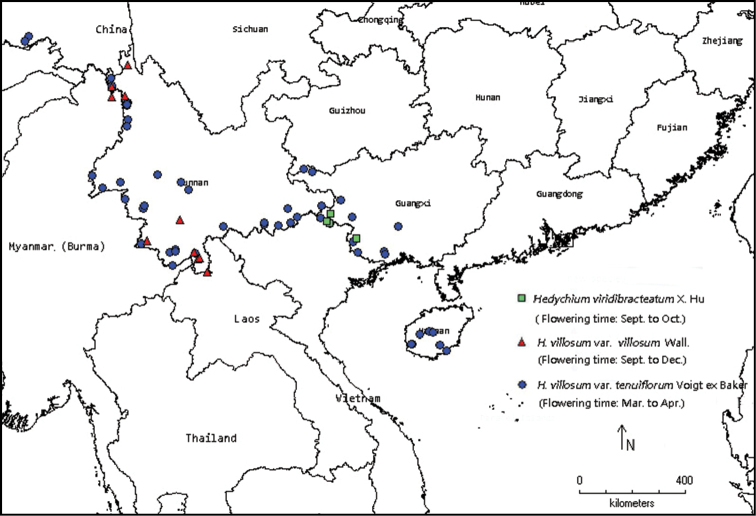
Distribution and flowering of *Hedychiumviridibracteatum* X.Hu, sp. nov. and the related species H.villosumvar.tenuiflorum and *H.villosumvar.villosum*.

In addition to the type location, the new species is found in the adjacent area, Longzhou and Jingxi Counties (Fig. 5). *Hedychiumviridibracteatum* can be easily distinguished from related species, even from dried specimens. The following sites were discovered by examining specimens and confirmed by our field investigations from 2012–2017: 20 September 1935, X. P. Gao 55777, Guangxi Autonomous Region, Jingxi County, Ande district (IBSC 0022836); 13 September 2007, W. B. Xu & Y. Y. Liang 0144, Guangxi Autonomous Region, Jingxi County, Bangliang National Nature Reserve (IBK 00223131); 29 September 2007, W. B. Xu & Y. Y. Liang B0065, Guangxi Autonomous Region, Jingxi County, Bangliang National Nature Reserve (IBK 00223137); 16 September 2010, Y. S. Huang et al. LYJX0450, Guangxi Autonomous Region, Jingxi County, Bangliang National Nature Reserve (IBK 00224854); 27 October 2010, W. H. Wu W0286, Guangxi Autonomous Region, Longhzhou county, Nonggang National Nature Reserve (IBK 00216857).

##### Other *Hedychium* spp. examined

**HedychiumvillosumWall.var.tenuiflorum Voigt ex Baker**

CHINA. **Guangxi**: Baise County: Daleng, 800 m, 26 Mar 1975, *D. Fang 36943* (GXMI); Longguang, 900 m, 14 Jan 1956, *Baise Group 1914* (IBK); Longlin County: Dee, Yanyu, 933 m, 9 Sep 1977, *J.F. Wei & D. Fang 3–1145* (GXMI); Longlin County: Zhelang, Hanshan, 1000 m, 24 Mar 1975, *D. Fang 25610* (GXMI); Longzhou County: Binqiao, Anzhen village, 273 m, 29 Apr 1956, *Y.K. Li 212* (IBK); Napo County: Pingmeng, Mengda, back Mountain, 600 m, 16 Apr 1976, *D. Fang 22291* (GXMI); Pingmeng, Nonghua to Sheng village, 700 m, 12 May 1989, *South China team 702* (IBSC); Pingmeng, Nongyi, Stone Mountain, 646 m, 1 Mar 2006, *M. Liu 3* (HITBC); Nanning County: Maoqiao, Medicinal Garden, 80 m, 3 Mar 1975, *D. Fang & X.X. Chen 78882* (GXMI); Shangsi County: Fenghuang, Longshan Mid-range, 811 m, 4 Mar 1944, *S.Q. Chen & S.H. Chun 4628* (IBSC); Fulongai, back valley, 607 m, 23 May 1966, *T.J. Wang 5393* (GXMI); Tiandeng County: Fuxin, Miao village, 502 m, 18 Sep 1977, *Tiandeng County Investigation Team 2–233* (GXMI); **Hainan**: Changjiang County: Bawang Mountain, Dongsi Forest Farm, 1400 m, 20 Apr 1988, *Z.X. Li & F.W. Xing 3526* (IBSC); Bawang Mountain, Dongwu Forest Farm, 1015 m, 27 Mar 1983, *G.Y. Fu 3418* (IBSC); Baoting County: Diaoluo Mountain, Xin’an village, 84 m, 24 Dec 1954, *Diaoluo Mountain Team 3280* (IBSC); Qizhi Mountain, 185 m, 9 Jan 1934, *Z. Huang & C. Wang 36223* (IBSC); Jianfeng Mountain, 830 m, 15 Apr 1982, *Q. Huang 820154* (IBSC); **Xizang**: Motuo County: Damu to Gedang, 850 m, 25 Mar 1993, *H. Sun & Z.K. Zhou & H.Y. Yu 5043* (KUN); **Yunnan**: Fugong County: Pihe, east coast of Nu River, 1100 m, 30 May 1978, *Bijiang Investigation Team 0257* (KUN); along Nu River, Stone Mountain, 1262 m, 9 Apr 2008, *F. Yu 19* (HITBC); Diao Ga Guo Zhai, 25 Apr 2004, *D. Heng 49837* (GH); Funing County: Banlun, 728 m, 10 Apr 1940, *Q.W. Wang 88283* (KUN); Gengma County: 1670 m, 1 Apr 1936, *Q.W. Wang 72918* (IBSC); along the Gengma River, 1280 m, 7 Mar 2008, *F. Yu 20* (HITBC); Gongshan County: Dulongjiang, Longyuan, 1772 m, 12 Apr 1994, *Dulongjiang Investigation Team 5582* (KUN); Hekou County: Dawei Mountain, Laozhai, 1338 m, 6 Mar 1940, *X. Wang & X.P. Gao & X.Q. Liu 100235* (IBSC); Qiaotou, 958 m, 1 Apr 2008, *F. Yu 21* (HITBC); Jingdong County: Dongchuan River, 1097 m, 26 Mar 1940, *M.G. Li 1900* (KUN); Wuliang Mountain, 1500 m, 19 Mar 2008, *F. Yu 22* (HITBC); Jinghong County: Damenglong Mengsong Mountain, 1800 m, 29 Nov 2006, *S.S Zhou 3409* (HITBC); Liuku County: Nu River, along the road, 1241 m, 8 Apr 2008, F. Yu 18 (HITBC); Mang County: Mengjiu, 1601 m, 28 Jul 1984, *Q.G. Wu 96* (IBSC); Lvchun County: Huanglianshan national Nature Reserve area, 1500 m, 28 Apr 1984, *S.Q. Dong & A.M. Li 24905* (HITBC); Malipo County: Huangjinyin, 1455 m, 13 Jan 1940, *Q.W. Wang 83189* (KUN); Nanwen River, Laojun Mountain, 1600 m, 1 Jun 1983, *S.Q Dong 32927* (HITBC); Menghai County: Mengsong, Manjin, 1221 m, 27 Mar 1957, *Zhongsu Team 5469* (IBSC); Mengla County: Menglun Tropical Botanical Garden, 564 m, 25 Jan 2007, *L.Q. He 119* (HITBC); Xiangming, 900 m, 26 Mar 1984, *S.Q. Dong & A.M. Li 24897* (HITBC); Pingbian County: 1677 m, 9 Mar 1934, *Y. Qian 13443* (IBSC); Yanshan County: Shuitouzhai, 1200 m, 19 Oct 1939, *Q.W. Wang 84527* (KUN); Yingjiang County: Tongbiguan, 1400 m, 9 Apr 1985, *Examination Team 85–200* (KUN); Zhenkang County: Bainiu village, 1593 m, Mar 1936, *Q.W. Wang 72237* (KUN).

**Hedychiumvillosumvar.villosum Wall**

CHINA. Yunnan: Fugong County: Maji Township, 1337 m, 4 Nov 1990, *Dulong River Investigation Team 140* (KUN); Gongshan County: Dulong River to Meilin River, 1400–1420 m, 11 Jul 1979, *Q. Lin & X.F. Deng 2731* (KUN); Dulong River East Coast, 2200–2400 m, 20 Sep 1940, *G.M. Feng 2168* (KUN); Dulong River West Coast, 1300 m, 20 Nov 1990, *Dulong River Investigation Team 637* (KUN); Dulong River West Coast, Four village, 1300 m, 25 Nov 1959, *G.M. Feng 24396* (KUN); Mengla County: Menglun, 800 m, 6 Dec 1972, *Y.H. Li 8466* (HITBC); Menglun, 26 Jan 1979, *J.Y. Cui 14683* (HITBC); Menglun, Silver Factory, limestone mountains, 860 m, 6 Jan 1960, *Y.H. Li 2731* (HITBC); Menglun, limestone mountains, 1200 m, 23 Nov 2005, *S.S. Zhou 3320* (HITBC); Mengyuan, Nov 1982, *Expedition team 34279* (HITBC); Shangyong, 900 m, 18 Oct 1974, *Z.H Yang 10983* (HITBC); Yiwu Dt.: Manpi, 1000 m, 8 Sep 1959, *S.J. Pei 9993* (HITBC); Menglian County: 957 m, 2 Oct 2006, *X. Hu 001* (IBSC).

## Discussion

*Hedychiumviridibracteatum* X.Hu is included in the short-anther group of *Hedychium*. It is similar to *H.villosum* in having visible rachis, non-imbricate bracts, more than one flower per bract, long filament and short, dorsifixed anther. It differs from H.villosumvar.tenuiflorum in having short green ligules (vs. long and pale pink), elliptic glabrous leaf blade (vs. elliptic-lanceolate, midrib pubescent), short green (vs. brown) bracts and bracteole, flowers without much fragrance (vs. mildly fragrant), green calyx (vs. pink tinged towards tip), pure white short floral tube and filament (vs. creamy-white floral tube and red filament), pure white corolla lobes (vs. greenish-white lobe), pure white lateral staminodes with dentate tip (vs. a yellow tinge at base, tip acute lateral staminodes), pure white ovate labellum, apex incised to the middle (vs. white with a yellow tinge at base oblanceolate labellum, apex deeply divided) blooming in September to October (vs. in March–April) (see Table 1).

**Table 1.** A morphological comparison of *Hedychiumviridibracteatum* with related species.

Characters	* H. viridibracteatum *	H. villosum var. villosum	H. villosum var. tenuiflorum
Ligule	Oblong-ovate, green or purple, 1.8–2.3 × 1.2–1.5 cm, glabrous	Oblong, pale pink, 1.8–2.1 × 0.7–0.8 cm, pubescent externally	Oblong, pale pink, 2.9–3.4 × 1.3–1.6 cm, densely pubescent externally
Leaf blades	Elliptic, purple beneath, 15–25 × 5–8 cm, glabrous on both sides.	Elliptic-lanceolate, pale green below, 14–20 × 3.7–4.5 cm, midrib pubescent below	Elliptic-lanceolate, pale green below, 34–40 × 8–9 cm, midrib pubescent below
Bract	Oblong-ovate, green, 1.3–1.5 × 0.4–0.5 cm, pubescent outside, 2–4 flowered	Elliptic, brown, 1.2–1.4 × 0.4–0.5 cm, densely hairy externally; 2–3–flowered	Lanceolate, brown, 2.7–2.9 × 1.1–1.2 cm, densely hairy externally; 3–4 flowered
Bracteole	Green, 1.0–1.1 × 0.3–0.4 cm, pubescent outside	Brown, 1–1.3 × 0.25 cm, densely hairy externally	Brown, 2.1–2.2 × 0.7–0.75 cm, densely hairy externally
Flowers	White, 10.0–11.0 cm long, lightly fragrant.	Pale yellow, 4.7–5.2 cm, highly fragrant	White with red stamen, 11.8–12.2 cm long, mildly fragrant
Calyx	Pale green, 2.5–2.8 × 0.12–0.15 cm, pubescent outside	Pale green, 1.6–1.8 × 0.15 cm, densely pubescent externally, hairs brown	Pale green, pink tinged towards tip, 3.3–3.7 × 0.2–0.3 cm, densely pubescent externally
Floral tube	Pure white 4.0–5.0 cm long,	Pale yellow, 1.9–2.1 cm long	Creamy-white, 5–5.2 cm long
Corolla lobes	Linear, pure white dorsal lobe 3.1–3.3 × 0.15 cm; lateral lobes 2.9–3.1 × 0.15cm	Oblong, pale yellow; dorsal lobe 1.6–1.7 × 0.15–0.2 cm; lateral lobes 1.5–1.6 × 0.15 cm	Oblong, greenish-white, dorsal lobe 4.3–4.4 cm long; lateral lobes 4–4.1 cm long
Lateral staminodes	Linear-ridged, pure white 3.0–3.3 × 0.2–0.4 cm, tip dentate	Linear, 1.3–1.5 × 0.15 cm; tip acute	Linear, white with a yellow tinge at base, tip acute, rarely forked 3.3–3.5 × 0.1–0.15 cm
Labellum	Ovate, pure white, 2.8–3.0 × 1.2–1.4 cm, base attenuate into 8–10×2 mm claw, apex incised to the middle	Elliptic, pale yellow, 1.3–1.4 × 0.5 cm, apex deeply divided (sinus 0.8–0.9 cm deep); claw 1 mm wide	Oblanceolate, white with a yellow tinge at base, 3.1–3.5 × 1.2–1.5 cm, apex deeply divided (sinus 1.9–2.2 cm deep; claw 4–4.5 mm wide
Filament	Pure white, 6.0–6.8 cm long	Scarlet, 2.4–2.6 cm long	Red, light red towards tip, 5.3–6.3 cm long
Anther	Light-yellow, 3.5–4 mm long	Brown, 1.5 × 1.5 mm long	Brown, 3.5 × 2.5–3 mm long
Flowering time	Sept–Oct	Sep–Dec	Mar–Apr
Ploidy	Tetraploid	Diploid	Tetraploid

Although *Hedychiumviridibracteatum* and H.villosumvar.tenuiflorum have the same ploidy level the two species differ in their flowering time which ensures that they are reproductively isolated. *Hedychiumviridibracteatum* shares the same flowering time with H.villosumvar.villosum, but they can be distinguished by their different flower form, flower size (Table 1), ploidy level (Fig. 4), and are geographically isolated (Fig. 5). The new species is also similar to the newly published *H.chingmeianum* N. Odyuo & D. K. Roy, 2017 (Odyuo and Roy 2017) but can be distinguished by having green bract (vs. purplish-brown), larger flowers (10.0–11.0 cm long vs. 4–5 cm long), a longer filament (6.0–6.8 cm vs. 2.8–3.0 cm) with white colour (vs. brownish-red). Thus, we confirm that *H.viridibracteatum* is a new species.

## Supplementary Material

XML Treatment for Hedychium viridibracteatum
